# Infectious hypothesis of Alzheimer disease

**DOI:** 10.1371/journal.ppat.1008596

**Published:** 2020-11-12

**Authors:** Charles E. Seaks, Donna M. Wilcock

**Affiliations:** Sanders-Brown Center on Aging, University of Kentucky, Lexington, Kentucky, United States of America; University of Kentucky, UNITED STATES

## Alzheimer disease

Alzheimer disease (AD) is the leading cause of dementia worldwide, accounting for almost 70% of all dementia cases, and is one of the fastest growing healthcare price burdens. Pathologically, AD is characterized by the presence of beta amyloid (Aβ) plaques and neurofibrillary tangles composed of aggregated microtubule associated protein tau. Clinically, AD manifests as progressive cognitive decline and worsening memory deficits [1]. The traditional hypothesis on the progression of AD pathologies states that Aβ plaques appear first, causing hyperphosphorylation of tau, leading to tangles and neurodegeneration. However, with the continued failure of clinical trials aimed at decreasing Aβ plaques, this hypothesis has come under scrutiny while alternative hypotheses are being investigated. One of the more controversial emerging hypotheses is the infectious hypothesis.

## The infectious hypothesis

**Fig 1:**
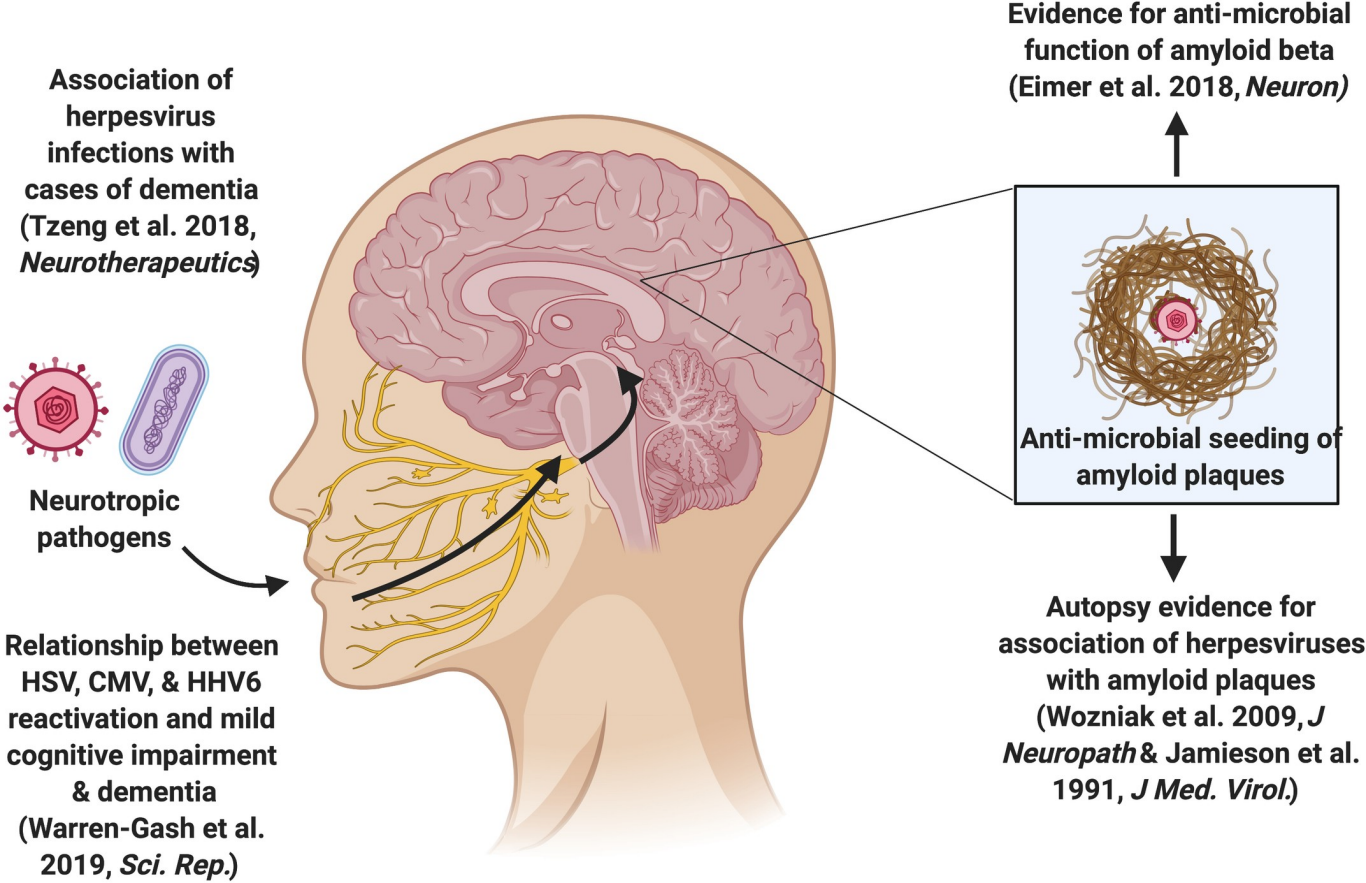
Conceptual framework of the infectious hypothesis as it relates to beta amyloid plaque deposition. *Created with Biorender.com*.

The infectious hypothesis proposes that a pathogen (virus, bacteria, prion, etc.) is the root cause of AD [2]. The hypothesis is supported by evidence that some pathogens, such as herpesviruses and certain bacterial species, are found more commonly in AD patients. There is some variation within the infectious hypothesis field as to how an infectious pathogen explains the pathological hallmarks of AD. Direct infection and eventual death of central nervous system (CNS) cells by pathogens could explain the cognitive deficits and heightened inflammation found in AD [3]. The relationship between inflammation and the AD hallmarks has long been recognized, with inflammation hypothesized to cause tissue damage, leading to protein aggregates such as Aβ plaques and tangles, which in turn can lead to more inflammation [4]. This cascade could be initiated by a number of endogenous and external factors, including microbial pathogens. Alternatively, Aβ and tau may be the products of normal responses to infection, intended to sequester threats to the CNS [5]. Accumulation of Aβ and tau could then occur when the generation of the aggregates outpaces clearance by the microglia in the brain, a process brought about by the natural process of aging [5]. Such an antimicrobial role–centered model is illustrated in Fig 1. The aggregates themselves have shown to trigger neuroinflammation as well [6]. Recent findings have highlighted a number of pathogens as potential drivers of AD, but the family of pathogens most investigated is the herpesviruses [7].

## Herpesviruses and potential contributions to Alzheimer disease: What is currently known?

The herpesviruses are a family of enveloped, double-stranded DNA viruses capable of infecting a wide range of mammalian species [8]. The worldwide ubiquity of herpesviruses tends to be high due to their relatively broad tropism, with some species, such as the herpes simplex viruses, reaching over 80% seroprevalence in humans [7]. Herpes simplex virus 1 (HSV-1) is the most commonly studied pathogen in the context of AD, primarily due to identification years ago of HSV-1 DNA in AD patient brains at autopsy [9,10]. The first of these studies [10] identified latent virus in both normal and AD brains but postulated that differences in viral expression and susceptibility might underlie HSV-1 contribution to AD. Itzhaki and colleagues [9] then demonstrated that the presence of ApoE4, the strongest genetic risk factor for AD, and HSV-1 together was a stronger risk factor for the development of AD than either factor on its own. While a role for HSV-1 has long been suspected in AD, other members of the herpesviruses have recently been implicated, including cytomegalovirus, Epstein–Barr virus, and human herpesvirus 6 (HHV6) [11]. Largely, these studies have consisted of screening patient samples for the presence of these viruses, either through immunoglobulin titers or direct PCR amplification of viral DNA, and then comparing between AD and normal patients. More specific work has focused on HHV6’s ability to seed Aβ plaques in vitro and in vivo [12].

HSV-1 establishes latency in the ganglia of the trigeminal nerve, giving it access to the CNS after initial infection in the peripheral tissue of the lips and mouth [8]. Latency of HSV-1 is maintained by an RNA transcript known as the latency transcript [13]. This prevents the host immune system from identifying mature viral proteins and nucleic acids that would trigger an innate immune response [8]. This latency can be broken by a number of factors, including UV light, physical damage, hormonal changes, and off-target effects of pharmaceuticals, leading to a reestablished active infection [13]. The chief clinical indicator of this reactivation is the presence of the herpetic lesions colloquially known as “cold sores” or “fever blisters.” In a healthy individual, the infection remains in the periphery, as the intrinsic immunity of the brain prevents the further propagation of the virus into the CNS [14]. However, in older or immunocompromised individuals, this intrinsic immunity is impaired, allowing the virus to spread into the rest of the brain [15].

The presence of herpesviruses in the CNS may clinically progress in a number of ways. The most severe, but relatively rare, outcome is an exaggerated immune response leading to herpes simplex encephalitis [16]. The potentially more relevant course for development of AD is a subclinical infection resulting in the virus establishing latency in CNS resident cells [17]. From here, the virus can reactivate periodically, spreading further and establishing more latent populations. This cycle of active and latent states could also explain the aging nature of AD, with a certain number of reactivation cycles to reach a viral load level necessary to overwhelm the response of the brain or cause enough damage to exhibit clinical symptoms [17]. De Chiara and colleagues demonstrated that recurrent reactivation of HSV-1 in a wild-type mouse model could produce hallmark AD pathology, accompanied by cognitive deficits, an intriguing result that supports the hypothesis that reactivation is critical in the connection between herpesviruses and AD.

Herpesviruses have been implicated in AD through the isolation of viral DNA from AD patients, as well as through epidemiological data [18]. A relatively recent study utilizing national insurance information in Taiwan showed that patients showing infection by herpesviruses were more likely to be diagnosed with multiple forms of dementia, including AD. Specifically, patients infected with HSV-1 had a hazard ratio of 2.564, indicating a 2.56-fold increase in likelihood of developing dementia [18]. Additionally, they showed that treatment with antiherpetic therapeutics, specifically acyclovir, decreased the likelihood of being diagnosed with those same forms of dementia significantly. Use of any antiherpetics resulted in hazard ratios well below 1.0, indicating decreasing risk of developing dementia.

## Future directions of the infectious hypothesis

There is mounting support and evidence for the infectious hypothesis. At numerous conferences on AD, the number of abstracts examining the connection between pathogens and the development of AD has increased in recent years, and dedicated sessions and panels have begun to arise. Considerable work is still needed, however, to more convincingly connect AD and infectious agents. First and foremost, important questions on the relationship between established risk factors of AD and pathogen infections remain to be addressed. There has always been the lingering question of how pathogens ubiquitous in much of the population are causing a disease in only a subset of that population. The answer to this selective vulnerability likely lies within the infectious hypothesis’s interactions with other theories and risk factors. Could clearance of Aβ and tau, used to stop HSV-1 spread, be impaired due to changes in vascular health? Could breakdown of the blood–brain barrier with age be providing new sites of entry for other pathogens? There is therefore a need for increased research in the AD field with respect to infectious pathogens. Many of the well-known AD risk genes, such as apolipoprotein-E, are related to immunity or, in some cases, life cycle of the pathogens [11]. A review by Dr. Steven Harris and Dr. Elizabeth Harris discussing what we already know about these interactions is recommended to any interested readers (see [13]). Instead of viewing the infectious hypothesis as a standalone entity, it should be investigated through the lens of interactivity with other components of the disease. Such holistic, inclusive approaches are a natural progression toward a larger hypothesis, a process that the Aβ hypothesis has had to undergo, which then leads to another question: why has the process been so slow for the infectious hypothesis?

## The Alzheimer disease field and infectious hypothesis

The answer likely stems from the composition of the AD field. Though expanding and diversifying, most AD researchers are not microbiologists or virologists. Typically, they are neuroscientists, biochemists, neuropathologists, neuropsychologists, and pharmacologists. There is a distinct lack of overlap between the disciplines that is sometimes hard to bridge in terms of forming collaborations or productive dialogue. The number of individuals who work on AD and identify as a microbiologist or virologist is extremely small.

The longstanding support for Aβ research is overwhelming, despite high failure rates for therapeutics centered on Aβ modulation [19]. NIH funding for AD research as of this article’s writing sits at $2.3 billion, a funding level reflective of the imminent health concern AD represents. Of the NIH AD funding, a negligible amount is used to investigate pathogens in AD [20]. However, the recent announcement of special interest by the National Institute on Aging (NIA) for grants examining a potential connection between pathogens and AD represents an important step in increased funding in this area (Notice #: NOT-AG-19-012).

Despite the challenges the infectious hypothesis faces, there are reasons to be hopeful for further research in this area. Recent studies have contributed significant evidence to the infectious hypothesis and have received public attention [12,21]. Eimer and colleagues [12], as previously mentioned, demonstrated the ability of herpesviruses to seed Aβ plaques and showed that these plaques act in an antiviral manner. This provided a clear mechanistic connection between herpesviruses and the development of pathology. Readhead and colleagues [21] showed significant overlap of affected pathways in HHV6 infection and AD, specifically related to amyloid precursor protein (APP) processing to the Aβ peptide, forming oligomers, and ultimately amyloid plaques. This connection in pathways strongly supports the theory that herpesviruses contribute to AD and fits within the antimicrobial Aβ theory, with the presence of herpesviruses triggering up-regulation of APP metabolism.

Organizations related to the infectious hypothesis have been growing as well, including the Alzheimer’s Germ Quest, Inc, founded by Dr. Leslie Norins to provide a challenge award for research identifying a causative pathogen of AD. Representatives from the Human Herpesvirus 6 Foundation have been offering assistance in the form of reagents and funding for groups interested in pursuing the connection of HHV6 to AD. A petition for increased allocations of funds for infectious AD–related research has also been started, and funding opportunities, such as those provided by the NIA, will provide opportunities for further research. Ultimately, the opportunity for meaningful infectious hypothesis research has never been better. Alternative hypotheses of AD are being considered more openly than at any point in the past, given trial failures, and longtime researchers in the field have been fighting for opportunities that are now becoming a reality.

The next stage in infectious hypothesis research is difficult to predict. Recent work has highlighted the connection between HSV-1 reactivation and AD pathology [7]. Clinical trials are under way by researchers that are looking at the use of antivirals in treating AD (ClincialTrials.gov Identifier: NCT03282916). Gingivitis and chlamydia have also been recently implicated in AD, giving credence to the idea that Aβ plaques are a broad antimicrobial peptide [22,23]. Determining common characteristics of pathogens that contribute to AD would be a step forward, as it would allow investigators to identify other potential pathogens and winnow the field of potentially relevant interactions with risk factors. There are a large number of identified risk factors for AD, and establishing how pathogens may be interacting with them is an important issue. Ultimately, these questions require answers that will come slowly; however, increased attention, collaboration, and funding will be necessary to answer them.

For those with greater interest in specific aspects of the infectious hypothesis, we would direct you to more comprehensive reviews written by Dr. Ruth Itzhaki, who frequently publishes scientific reviews on the topic.
